# Emergency Debulking of Recurrent Respiratory Papillomatosis: An Anesthesia Challenge

**DOI:** 10.7759/cureus.74404

**Published:** 2024-11-25

**Authors:** Amit Padvi, Namita Padvi

**Affiliations:** 1 Anesthesiology, Al Jalila Children’s Hospital, Dubai, ARE; 2 Anesthesiology, Emirates Hospital, Dubai, ARE

**Keywords:** airway, anaesthesia, distress, emergency, hoarsness, papilloma

## Abstract

Recurrent respiratory papillomatosis (RRP) is a rare condition involving the recurrent growth of benign papillomas in the respiratory tract caused exclusively by human papillomavirus (HPV). We present the case of a five-year-old child who arrived at the emergency department with severe respiratory distress, hoarseness, and biphasic stridor. The patient required urgent transfer to the operating room for the emergency debridement of papillomas. Managing emergency pediatric airway obstruction poses significant anesthetic challenges and requires specialized skills and dedicated pediatric tertiary care facilities.

## Introduction

Recurrent respiratory papillomatosis (RRP) is a rare disorder marked by recurring benign papillomas in the respiratory tract caused by human papillomavirus (HPV) type 6 and 11. The disease predominantly affects the larynx, often resulting in dysphonia (voice changes), respiratory obstruction, tracheal and bronchial spread, emotional and psychological impact on the child, and other potentially serious complications. RRP can lead to significant morbidity, with complications arising from recurrent airway obstruction that may necessitate repeated surgical interventions [1]. In children, RRP is generally acquired perinatally, often linked to maternal HPV infection, whereas adult-onset cases are less commonly attributed to perinatal exposure and may involve different disease dynamics and risk factors [2]. Pediatric cases tend to present more aggressively, sometimes requiring frequent surgical debulking procedures to maintain an open airway, with significant impacts on quality of life. The larynx is the most frequently affected site, leading to substantial airway obstruction and potentially life-threatening complications [3]. In this case, we present a child with a known history of RRP who presented to the emergency department with acute airway compromise. Given the severity of the obstruction, an emergency surgical intervention was necessary to restore patency to the airway and prevent further complications. This case underscores the potential for sudden and life-threatening exacerbations in RRP patients, emphasizing the need for ongoing surveillance and rapid intervention strategies for those at risk.

## Case presentation

A five-year-old child with a prior diagnosis of RRP arrived at the emergency department in severe respiratory distress with a hoarse voice and biphasic stridor. Her symptoms included cough and difficulty breathing, which began two days before presentation. Upon arrival, the patient had resting stridor, tracheal tugging, and nasal flaring, with oxygen saturation (SpO₂) of 90-92% on room air, requiring supplemental oxygen 6 L/min. Bedside flexible laryngoscopy by the ENT surgeon revealed a significant recurrence of respiratory papillomas, necessitating urgent debridement in the operating room [4].

Preoperative assessment

The patient, weighing 16.5 kg, was classified as American Society of Anesthesiologists Grade 3 Emergency (ASA 3E) because of her respiratory distress and biphasic stridor. She presented with a hoarse voice and required high-flow oxygen through a non-rebreather mask at 10-15 L/min, achieving higher FiO_2_ (up to 90-100%) to maintain SpO_2_ at 95-96%. Her vital signs included a heart rate of 144 bpm and a respiratory rate of 34 breaths per minute. Chest auscultation was clear, though conducted sounds were noted due to stridor. Examination of the airway revealed posterior pharyngeal erythema. Due to the clinical condition, the patient fulfilled fasting criteria and had poor feeding [5].

After discussions with the ENT team, an emergency debridement procedure was planned under spontaneous ventilation using total intravenous anesthesia (TIVA). Informed high-risk consent was obtained from the patient’s parents, and the operating room was prepared with a difficult airway cart and emergency resuscitation cart. The ENT team set up for suspension laryngotracheal bronchoscopy with a tracheostomy kit on standby [6].

Intraoperative management

**Figure 1:**
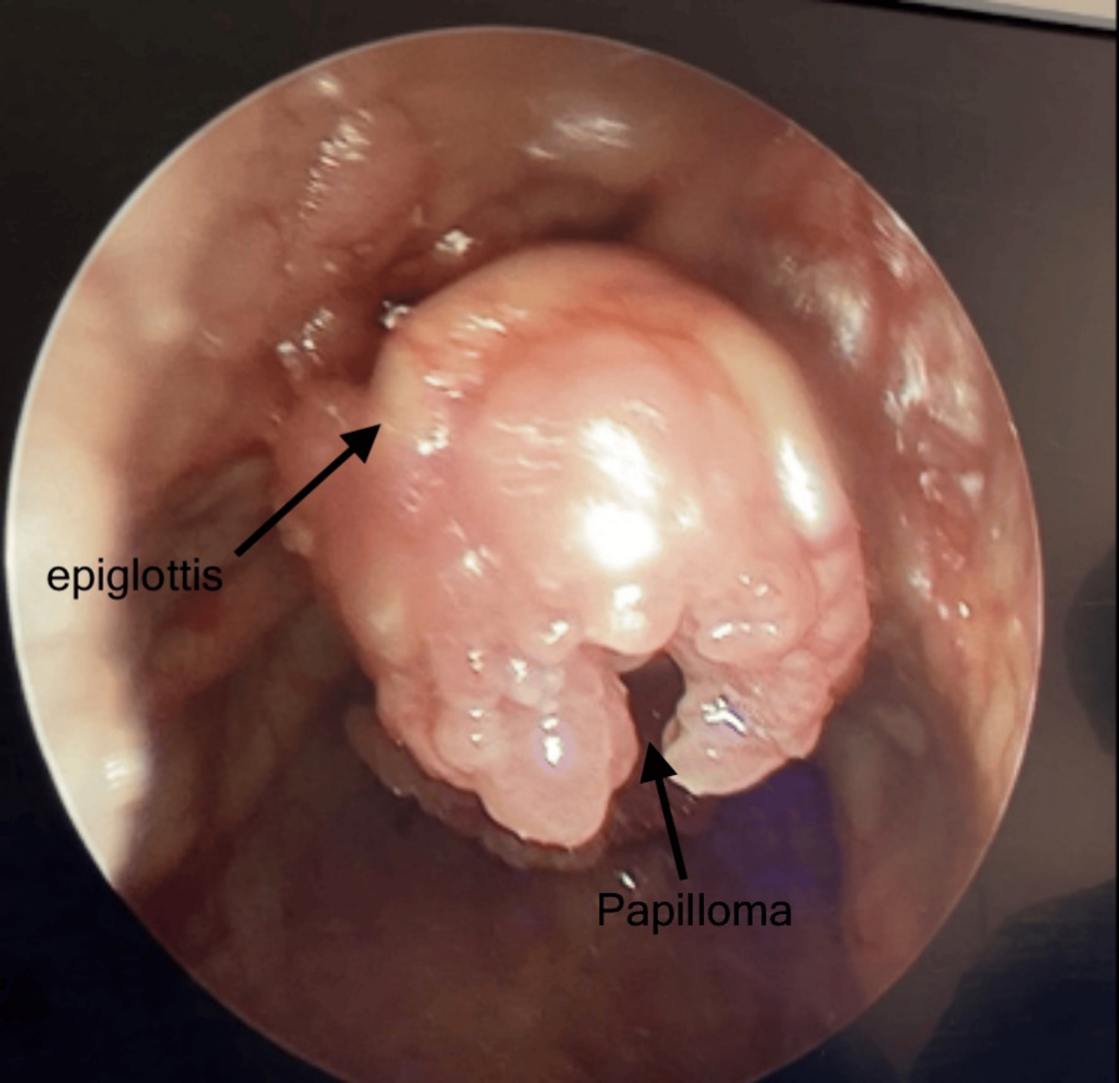
Epiglottis completely invaded with papillomas.

In the operating room, the patient was attached to standard ASA monitors. The patient secured IV access in the emergency room before shifting to the operation theater. The child was preoxygenated and premedicated with an injection of glycopyrrolate 60 mcg; sedation was initiated with 15 mcg of fentanyl while spontaneous ventilation was maintained. Anesthesia was deepened for direct laryngoscopy (Figure 1) and surgical intervention using a continuous infusion of remifentanil at 0.2 mcg/kg/min and propofol at 10 mg/kg/h. Oxygen was delivered through an auxiliary oxygen flow of 2 L/min connected to the suspension laryngoscope [7].

**Figure 2:**
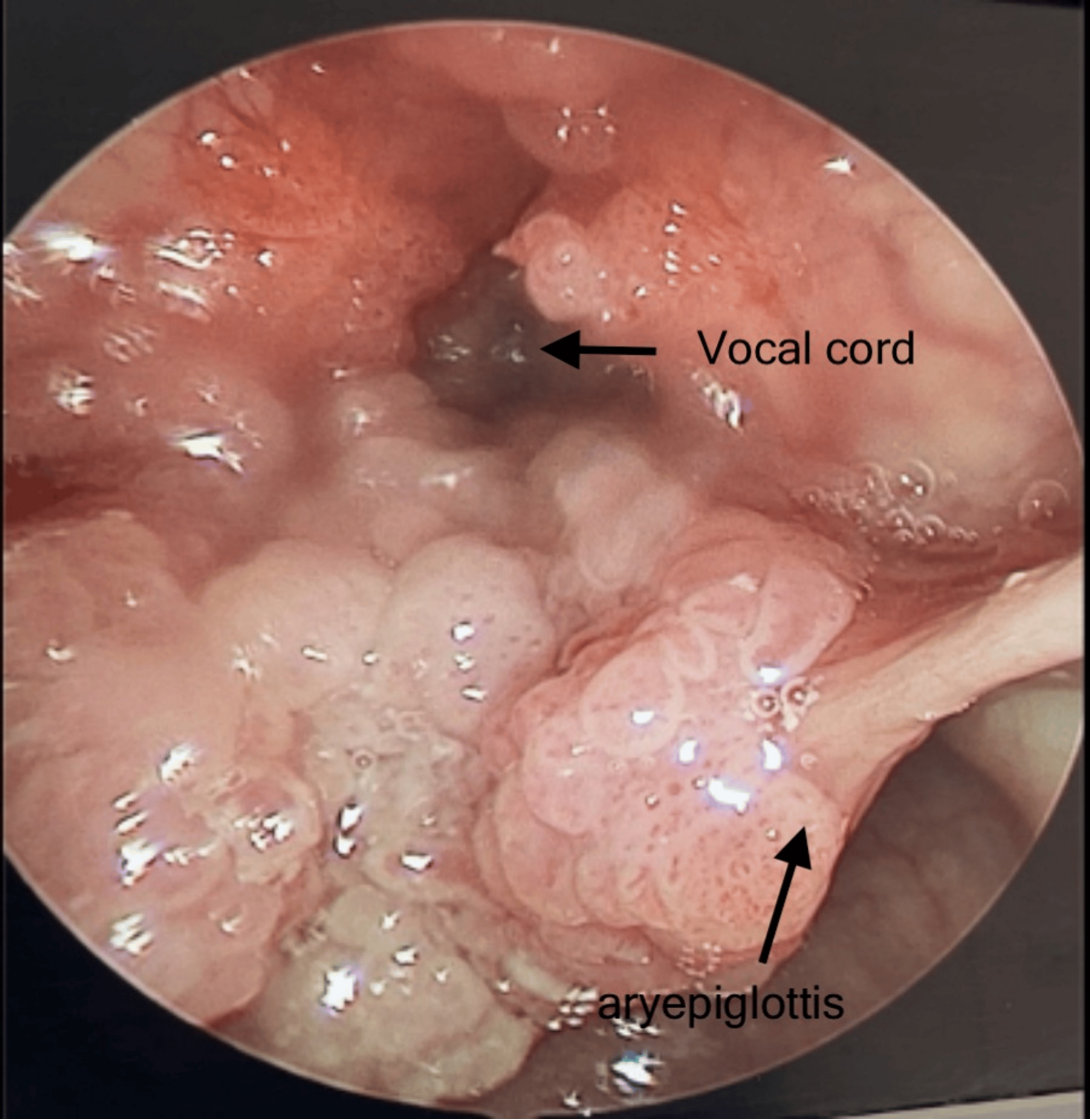
Extensive papillomas over aryepiglottic folds, ventricles, false and true vocal cords, posterior commissure.

Using a skimmer blade, extensive papillomas were debrided from the epiglottis, aryepiglottic folds, ventricles, false and true vocal cords, posterior commissure, and post-cricoid area (Figure 2). Spontaneous breathing was maintained throughout the procedure, and analgesia was managed by intermittent fentanyl boluses, injection of paracetamol 15 mg/kg, and a continuous remifentanil infusion [8]. Her vitals remained stable, with SpO_2_ between 98% and 99%, and no critical event occurred perioperatively. Following this, the patient was intubated with a size 4 micro cuff endotracheal tube, and an injection of dexamethasone 0.5 mg/kg was given.

Postoperative course

Postoperatively, the patient was transferred intubated to the pediatric intensive care unit (PICU) for close monitoring while intubated. She was successfully extubated two days later, and her recovery was uneventful. After one additional day of observation, she was discharged to the ward and subsequently sent home in stable condition.

Medical history

The patient first presented to our hospital’s ENT clinic at six months of age with progressive dysphonia, ultimately leading to near aphonia. Her parents had previously sought care at multiple hospitals, where she was initially misdiagnosed with vocal cord polyps or nodules. Examination revealed marked hoarseness without respiratory distress [9] or stridor, although she exhibited snoring. Initial laryngoscopy identified an enlarged, non-obstructive adenoid and numerous small lesions on both vocal cords, including larger polypoid lesions at the posterior glottic chink. Vocal cord movement was good despite a narrowed glottic opening.

The diagnosis of RRP was confirmed, and the patient underwent her first microlaryngoscopy, rigid bronchoscopy, and excision of papillomas from both vocal cords, extending to the anterior and posterior commissures. Although small papillomas were observed in the trachea, the carina and mainstem bronchi were clear. Debridement was performed to avoid scarring, and HPV infection was confirmed via pathology. She later underwent a second laryngoscopy with microdebrider excision of new papillomas in the hypopharynx, clearing the left vocal cord and ventricle of the disease [10].

## Discussion

Recurrent respiratory papillomatosis (RRP) is a rare, persistent disease caused by the human papillomavirus (HPV) [11], primarily affecting children aged two to four years. Its incidence in the pediatric population is estimated to be around 4.3 per 100,000 children. The condition commonly presents as hoarseness in young patients, with symptoms potentially progressing to severe respiratory complications, including life-threatening airway obstruction. Diagnosis can be challenging because RRP symptoms may resemble other respiratory conditions such as croup or asthma. The treatment of RRP usually involves surgical procedures aimed at alleviating airway obstruction and preserving vocal cord function. CO_2_ laser surgery and micro laryngoscopy are commonly employed techniques, although these often require repeated interventions due to the disease’s high recurrence rate.

Adjuvant therapies, including interferon, acyclovir, and intralesional cidofovir injections, have been explored to manage recurrence, but no definitive cure exists [12]. Surgical complications, such as airway scarring, edema, and stenosis, are frequently observed. A thorough preoperative assessment is essential to evaluate the degree of airway obstruction. This assessment typically includes evaluations of voice quality, respiratory status, heart rate, accessory muscle usage, and oxygen saturation. In cases where stridor is present, significant laryngeal obstruction is indicated, and flexible laryngoscopy performed by a skilled otorhinologist is necessary to confirm the diagnosis. For patients with suspected pulmonary hypertension due to prolonged respiratory obstruction, an echocardiogram may be warranted. Anesthesia management for RRP requires meticulous planning because the anesthesiologist and surgeon share access to the airway. The main objectives are to maintain adequate ventilation, ensure vocal cord relaxation, minimize trauma, and prevent laryngospasm, all while providing sufficient surgical access. Tracheostomy is typically avoided due to concerns over viral dissemination, with early decannulation prioritized if tracheostomy is performed. Several ventilation strategies are employed during laryngeal surgeries, including spontaneous ventilation, intermittent positive pressure ventilation, apneic ventilation, and jet ventilation. Total intravenous anesthesia (TIVA) with spontaneous ventilation and the use of small tubes may be appropriate in milder cases. For more severe cases, intubation offers control over the airway and reduces the risk of aspiration, although it can restrict surgical access and potentially spread the disease. To avoid obstruction of the endotracheal tube by RRP lesions, muscle relaxants should not be used until the airway is secure and spontaneous ventilation is maintained throughout the procedure.

## Conclusions

Management of RRP cases is often challenging due to the recalcitrant nature of the papillomas, which can recur even after complete surgical removal. Adjuvant therapies, including antiviral agents, bevacizumab, and photodynamic therapy, are being explored to reduce recurrence and improve patient outcomes, although their efficacy varies. Effective anesthetic management in RRP cases involves close coordination among the anesthetist, surgeon, and operating room team to achieve optimal care, especially given the risk of complete airway obstruction in young patients with narrow airways.
